# Risk factors during pregnancy and delivery for the development of Perthes’ disease, a nationwide Swedish study of 2.1 million individuals

**DOI:** 10.1186/s12884-020-2849-7

**Published:** 2020-03-30

**Authors:** Maria Lindblad, Ann Josefsson, Marie Bladh, Gunilla Sydsjö, Torsten Johansson

**Affiliations:** 1 Department of Orthopedics, Norrköping, Sweden; 2 Department of Biomedical and Clinical Sciences, Linköping University, Linköping, Sweden; 3 Department of Obstetrics and Gynecology, Linköping University, Linköping, Sweden; 4 Department of Biomedical and Clinical Sciences, Linköping University, Linköping, Sweden

**Keywords:** Birthweight, Breech position, Perthes’ disease, Preterm

## Abstract

**Background:**

To ascertain or disprove a correlation between suboptimal birth characteristics, breech position at delivery and development of Perthes’ disease.

**Methods:**

Study material was collected from nationwide registers regarding diagnoses, birth statistics and delivery data. As study population were included children with a diagnosis code for Perthes’ disease who were alive and living in Sweden at age 13. Children with missing birth statistics were excluded. All children with no Perthes’ disease diagnosis were used as control group. Both single and multiple logistical regression analyses were used to calculate OR for the included characteristics.

**Results:**

Children in breech position had a higher risk for developing Perthes’ disease. Children with Perthes’ disease had also a higher probability of having been born pre-term, very pre-term or post-term. Lower than normal birth weight and a lower Apgar-score were also associated with Perthes’ disease.

**Conclusions:**

There is a correlation between breech birth and development of Perthes’ disease. There is also correlation to suboptimal birth characteristics. Despite our findings this should not be used for screening of Perthes’ disease as the percentage of children who actually develop it is very low. Also, as of yet there is no possibility to diagnose Perthes’ disease before the presence of skeletal changes. Our findings could be important in finding the cause of Perthes’ disease and therefore developing better diagnostics, treatment and prevention.

## Background

Perthes’ disease or Legg-Calvé-Perthes disease was first described in 1910. It is defined as an osteonecrosis of the femoral head usually diagnosed during childhood, and it is 3–4 times more common in boys than in girls [1]. The most common symptom is pain that can be experienced in the hip, but also in the groin or knee. The child usually has a limp, which can be present even without pain. The abduction and internal rotation may be affected. The osteonecrosis leads to varying degrees of deformity in the femoral head, which can increase the risk for osteoarthritis later in life.

In a review including 21 studies from 16 countries, the incidence of Perthes’ disease ranged from 0.2 per 100,000 to 19.1 per 100,000 [2]. The reported annual incidence in a recent Swedish study was 9.3 per 100,000 subjects, and the ratio of boys to girls was 3.1:1 [1].

The etiology of Perthes’ disease is unknown but several correlations and theories have been presented. Ethnicity seems to be a factor, with Caucasian persons having a higher risk for Perthes’ disease. It is unclear if this is related to genetics or environmental factors [1, 2]. The risk for Perthes’ disease increases with more northerly geographical locations [2]. Perthes’ disease also seems to be more common in lower socioeconomic groups [1, 3].

Another hypothesis is that Perthes’ disease is vascular in origin [4]. Kemp reported, from an experimental study, that increased intracapsular pressure of the hip joint in dogs produced changes identical to Perthes’ disease in humans [5]. It is therefore possible that a long-standing breech position during fetal life and also being born with a breech presentation may prevent optimal circulation in the hip, leading to risk for later disease. Wynne-Davies and Gormley found in their study in 1978 that 1 in 10 children with Perthes’ disease had been a breech birth, transverse lie, or had had a version late in the pregnancy [4]. Also, 3% of all pregnancies are in breech position after 37 weeks. There are no studies as of yet regarding how many of these children develop Perthes’ disease later on. Studies show that vaginal breech delivery increases the risk for mortality and morbidity in the child, whereas cesarean section is safer for the child [6, 7].

A lower birthweight has been seen in children with Perthes’ disease [4, 6]. In 2006, Wiig et al. found that a statistically significant proportion of the studied children were shorter in stature than average [8]. Low birth weight could be a sign of intrauterine growth restriction, which is a risk factor for breech presentation. Preterm children often have a lower birth weight than normal, and also have a higher risk for breech presentation [9]. Breech position is a risk factor for umbilical cord prolapse [10]. This in turn can lead to lack of oxygen and correlated damage. Maternal diabetes on the other hand is a known cause for polyhydramnios, which increases the risk for breech presentation.

Smoking during pregnancy increases intima-media thickness during early life, which is an indicator for damage in blood vessels. There is also a correlation with low birth weight and impaired fetal growth. In Bahmanyar’s study maternal smoking increased the risk for Perthes’ disease with 67% (OR 1.67, 95%CI 1.41–1.97). There was also a dose-dependent trend [6], i.e. the risk increased with a higher consumption of cigarettes.

It is important to further investigate possible pregnancy- and delivery- related risk factors for Perthes’ disease in a large population. Hence, the aim was to investigate pregnancy and delivery outcomes of all children with Perthes’ disease born in Sweden and compare to children without Perthes’ disease born during the same time period.

## Methods

We used national population-based registers, which enabled us to perform a national cohort study as well as a case-control study, the latter to provide validation of the findings from the cohort study. The reasoning behind this choice of analyses was that the incidence of Perthes’ disease is quite low and thus the numbers of children affected will be low, even in a national cohort study, while those without the presence of Perthes’ disease form a very large group. This may result in findings that are statistically significant but without clinical relevance [11]. The data was originally extracted for a previous study and re-analyzed for the purpose of this study [12–16].

### Registers

Data were collected from several population-based registers and linked using the unique personal identification number assigned to every person residing in Sweden. Registers included in the study were: The Medical Birth Register [12], which contains medical information regarding the mother’s diagnoses during pregnancy, delivery and the child’s postpartum characteristics; The National Patient Register [13, 14], which contains data on all inpatient and outpatient visits, such as age, sex and diagnoses; The Total Population Register [15], which contains information about the country of birth and migration for the patients’ parents: and The Cause of Death Register [16], which contains information on cause and date of death for all persons who die in Sweden. All these registers are of good quality and are regularly evaluated [12, 14, 17–20].

Children were identified as having been diagnosed with Perthes’ disease if they had received the following diagnoses: ICD-8722, 10–722 and 11,722, 19; ICD-9732B and ICD-10 M911, M912 and M913. These children served as the cases.

### Cohort study

**Fig. 1:**
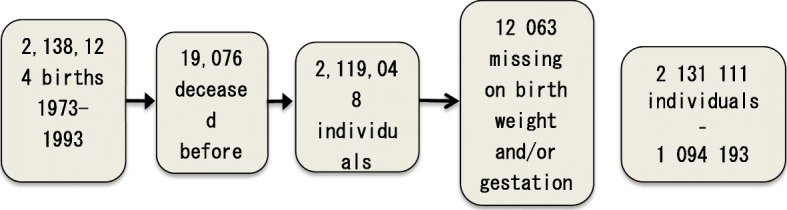
Flow chart showing the “cleaning” of the study population

The study population consisted of all children born in the period 1973–1993, alive and living in Sweden at 13 years of age. In setting up the data, individuals who were deceased before their 13th birthday, who did not reside in Sweden at the age of 13, or with missing values on birth weight and/or gestational length were removed. The final study population consisted of 2,131,111 individuals, of which 1,094,193 were boys and 1,036,918 were girls, and they were followed until 2013. Among these individuals, 1987 were identified to have been diagnosed with Perthes’ disease. (Fig. 1).

### Case-control study

The 1987 cases were compared to a control group of 3974 individuals without Perthes disease. The controls were randomly selected from the cohort study and matched for age and gender.

### Definitions

Large for gestational age (LGA) > + 2 SD of the mean weight for the gestational length according to the Swedish standard [21]

Small for gestational age (SGA) < − 2 SD of the mean weight for the gestational length according to the Swedish standard [21]

Low birth weight Birth weight below 2500 g

Very low birth weight Birth weight below 1500 g

Preterm Born between gestational week 32 + 0 and 36 + 6 gestational weeks

Very preterm Born before 32 + 0 gestational weeks

Diabetes Mothers with a diagnosis of any type of diabetes during pregnancy

Hypertension Mothers with a diagnosis of any type of hypertension during pregnancy

Breech delivery The child was positioned with its buttocks towards the birth canal during delivery

Apgar 5 Apgar-score recorded 5 min after birth.

### Statistical methods

An initial analysis of group differences on pregnancy, delivery, and birth characteristics was performed using Pearson’s chi-square. Individuals diagnosed with Perthes’ were compared with the cohort population and the matched controls in two separate analyses. Single logistical regression analyses were performed in order to calculate odds ratios (ORs) for Perthes’ disease in relationship to pregnancy, delivery, and birth characteristics, including the following variables: birth weight for gestational age, birth weight, gestational age, Apgar at 5 min, diabetes, hypertension and mode of delivery. Gestational age and birth weight were added as covariates in the multiple logistic regression models to adjust for their potential impact on OR. These analyses were performed in two steps, one for the entire study population and another for the case-control generated group. ORs were presented with 95% confidence intervals. All statistical analyses were performed using IBM SPSS, version 23 (IBM SPSS Inc., Armonk, NY).

### Ethics

This study was approved by the Regional Ethics Committee in Linköping (entry no. 2010/403–31).

## Results

In Table 1, a general description of the study populations and the pregnancy, delivery and birth variables are presented (total numbers and percentages).

**Table 1:** Description of the study populations’ pregnancy- and delivery-related characteristics

	Cases	Cohort controls	Matched controls		
Birth characteristic	Perthes n (%)	Not Perthes n (%)	Not Perthes n (%)	p-value^a, c^	*P*-value^b,c^
**Diabetes during pregnancy**				0.781	0.310
No	1955 (98.4)	2,096,467 (98.5)	3923 (98.7)		
Yes	32 (1.6)	32,657 (1.5)	51 (1.3)		
**Hypertension (mother)**				0.769	0.460
No	1965 (98.9)	2,104,036 (98.8)	3921 (98.7)		
Yes	22 (1.1)	25,088 (1.2)	53 (1.3)		
**Vaginal delivery (breech)**				0.014	0.750
No	1841 (92.7)	2,000,647 (94.0)	3691 (92.9)		
Yes	146 (7.3)	128,477 (6.0)	283 (7.1)		
**Emergency cesarean section (breech)**				0.010	0.024
No	1956 (98.4)	2,108,099 (99.0)	3938 (99.1)		
Yes	31 (1.6)	21,025 (1.0)	36 (0.9)		
**Elective cesarean section (breech)**				0.092	0.229
No	1979 (99.6)	2,124,355 (99.8)	3965 (99.8)		
Yes	8 (0.4)	4769 (0.2)	9 (0.2)		
**Breech delivery**				< 0.001	0.141
No	1802 (90.7)	1,976,157 (92.8)	3649 (9.8)		
Yes	185 (9.3)	152,967 (7.2)	325 (8.2)		
**Birthweight for gestational age**				< 0.001	0.002
Appropriate for gestational age	1805 (90.8)	1,990,865 (93.5)	3698 (93.1)		
Large for gestational age	61 (3.1)	61,161 (2.9)	115 (2.9)		
Small for gestational age	121 (6.1)	77,098 (3.6)	161 (4.1)		
**Birthweight**				< 0.001	< 0.001
Normal birthweight, ≥2500 g	1865 (93.9)	2,045,135 (96.1)	3816 (96.0)		
Low birthweight, 1500 g – 2499 g	99 (5.0)	74,789 (3.5)	139 (3.5)		
Very low birthweight, < 1500 g	23 (1.2)	9200 (0.4)	19 (0.5)		
**Gestational age**				< 0.001	0.002
At term, 37–42 weeks	1630 (82.0)	1,818,175 (85.4)	3380 (85.1)		
Post term, > 42 weeks	205 (10.3)	196,834 (9.2)	383 (9.6)		
Preterm, 32–36 weeks	135 (6.8)	103,207 (4.8)	194 (4.9)		
Very preterm, < 32 weeks	17 (0.9)	10,908 (0.5)	17 (0.4)		
**Apgar 5**				< 0.001	0.013
> 7-max	1908 (97.2)	2,072,508 (98.2)	3862 (98.2)		
0–7	55 (2.8)	36,965 (1.8)	71 (1.8)		

^a^ cases vs population; ^b^ cases vs match controls; ^c^ Pearson’s chi-square

### Single logistic regression

This study showed that children diagnosed with Perthes’ disease had an overall higher risk of being born in a breech position (OR = 1.33, 95% CI = 1.14–1.54), and sub-analyses on mode of delivery among these revealed that children with Perthes’ had an increased risk of being born vaginally with a breech position (OR = 1.24, 95% CI = 1.04–1.46) and increased odds ratio for being born by emergency CS (caesarean section) in a breech position (OR = 1.59, 95% CI = 1.11–2.27) compared with controls (Table 2). Moreover, being diagnosed with Perthes’ disease was associated with an increased likelihood of being born small for gestational age (SGA) (OR = 1.73, 95% CI = 1.44–2.08), having either a low or very low birth weight (OR = 1.19, 95% CI = 1.19–1.78; OR = 2.74, 95% CI = 1.82–4.14, respectively), and of being born very preterm, preterm or post term (OR = 1.74, 95% CI = 1.08–2.80; OR = 1.46, 95% CI = 1.22–1.74; OR = 1.16, 95% CI = 1.01–1.34, respectively). Also, children diagnosed with Perthes’ disease had a lower Apgar at 5 min (OR = 1.61, 95% CI = 1.24–2.11) (Table 2). Restricting the analysis to the case-control study (as a way of evaluating the findings in the population study) most of these statistically significant increased OR remained (including: emergency cesarean section, SGA, low or very low birth weight, very preterm, and preterm delivery) (Table 2).

**Table 2:** Single and multiple regression analysis and odds ration with corresponding 95% CI of birth characteristics and delivery

	Population study	Case-control study	Population study	Case-control study
Birth characteristic	Perthes vs. not Perthes Single OR (95% CI)	Perthes vs. not Perthes Single OR (95% CI)	Perthes vs. not Perthes Multiple OR ^a^ (95% CI)	Perthes vs. not Perthes Multiple OR ^a^ (95% CI)
**Diabetes during pregnancy**				
No	Reference level	Reference level	Reference level	Reference level
Yes	1.05 (0.74–1.49)	1.26 (0.81–1.97)	1.05 (0.74–1.49)	1.25 (0.80–1.95)
**Hypertension (mother)**				
No	Reference level	Reference level	Reference level	Reference level
Yes	0.94 (0.62–1.43)	0.83 (0.50–1.37)	0.82 (0.53–1.25)	0.67 (0.40–1.12)
**Vaginal delivery (breech)**				
No	Reference level	Reference level	Reference level	Reference level
Yes	1.24 (1.04–1.46)	1.03 (0.84–1.27)	1.16 (0.98–1.37)	0.98 (0.79–1.21)
**Emergency cesarean section (breech)**				
No	Reference level	Reference level	Reference level	Reference level
Yes	1.59 (1.11–2.27)	1.73 (1.07–2.81)	1.52 (1.07–2.18)	1.64 (1.01–2.68)
**Elective cesarean section (breech)**				
No	Reference level	Reference level	Reference level	Reference level
Yes	1.80 (0.90–3.61)	1.78 (0.69–4.62)	1.78 (0.89–3.56)	1.86 (0.72–4.83)
**Breech delivery**				
No	Reference level	Reference level	Reference level	Reference level
Yes	1.33 (1.14–1.54)	1.15 (0.95–1.39)	1.25 (1.08–1.46)	1.09 (0.90–1.32)
**Birthweight for gestational age**				
Appropriate for gestational age	Reference level	Reference level	Reference level	Reference level
Large for gestational age	1.10 (0.85–1.42)	1.09 (0.79–1.49)	1.10 (0.85–1.42)	1.09 (0.79–1.49)
Small for gestational age	1.73 (1.44–2.08)	1.54 (1.21–1.96)	1.53 (1.22–1.91)	1.38 (1.03–1.84)
**Birthweight**				
Normal birthweight, ≥2500 g	Reference level	Reference level	Reference level	Reference level
Low birthweight, 1500 g – 2499 g	1.19 (1.19–1.78)	1.46 (1.12–1.90)	1.33 (1.05–1.69)	1.31 (0.97–1.76)
Very low birthweight, < 1500 g	2.74 (1.82–4.14)	2.48 (1.35–4.56)	3.32 (1.86–5.92)	2.20 (0.97–4.96)
**Gestational age**				
At term, 37–42 weeks	Reference level	Reference level	Reference level	Reference level
Post term, > 42 weeks	1.16 (1.01–1.34)	1.11 (0.93–1.33)	1.17 (1.01–1.35)	1.11 (0.93–1.33)
Preterm, 32–36 weeks	1.46 (1.22–1.74)	1.44 (1.15–1.81)	1.25 (1.01–1.54)	1.27 (0.98–1.64)
Very preterm, < 32 weeks	1.74 (1.08–2.80)	2.07 (1.06–4.07)	0.70 (0.36–1.36)	1.10 (0.45–2.73)
**Apgar 5**				
> 7-max	Reference level	Reference level	Reference level	Reference level
0–7	1.61 (1.24–2.11)	1.57 (1.09–2.24)	1.44 (1.10–1.90)	1.39 (0.97–2.00)

^a^ Odds ratio adjusted for gestational age and birthweight

### Multiple logistic regression

The multiple logistic regression analysis of the total population, where adjustments were made for gestational age and birth weight, showed that children diagnosed with Perthes’ disease had an increased odds ratio of being born in a breech position (OR = 1.25, 95% CI = 1.08–1.46), where a sub-group on mode of delivery showed an elevated likelihood of being born by emergency CS in breech position (OR = 1.52, 95% CI = 1.07–2.18) compared to children who had not been diagnosed with Perthes’ disease (Table 2). In addition, children diagnosed with Perthes’ disease were more likely to have been born preterm (OR = 1.17, 95% CI = 1.01–1.35) or post term (OR = 1.25, 95% CI = 1.01–1.54) and to have a lower Apgar at 5 min (OR = 1.44, 95% CI = 1.10–1.90) compared with controls. When the analysis was restricted to the case-control study the only remaining statistically significant increased odds ratios that remained were for being born SGA (OR = 1.38, 95% CI = 1.03–1.84) and being born by emergency CS in breech position (OR = 1.64, 95% CI = 1.01–2.68) (Table 2).

## Discussion

This study shows that children who were born in a breech position had an elevated risk of 25% for developing Perthes’ disease compared to children born in a non-breech position. In this study, we were unfortunately not able to confirm how long the children had been in breech position before delivery. This finding supports the results in the study by Wynne-Davies and Gormley [4] who found a strong relation between a malposition of the fetus and Perthes’ disease.

Children born with emergency CS in breech position had an elevated risk for developing Perthes’ disease. This elevated risk was not evident among children born with elective CS in breech position. Also, children that were born vaginally from a breech position had an elevated risk for development of Perthes’ disease. Nowadays, if a version is unsuccessful, it is common that children in a breech position are born by cesarean section if possible [21].

Our study confirms results from previous studies that have found a link between Perthes’ disease and low birth weight, SGA, as well as prematurity. These factors seem to be indicators of an elevated risk for future Perthes’ disease, and are likely to be related, as babies born preterm often also have a low birth weight. It should also be taken into consideration that the risk for breech position is higher in preterm deliveries [22]. Also, both premature birth and breech presentation are associated with a higher rate of birth abnormalities and disability [23, 24].

When discussing the possible causative connections between Perthes’ disease and elevated risk factors related to pregnancy and childbirth, it needs to be considered that these factors most probably are linked. It is quite possible that the reason for breech presentation is in fact more important for the etiology of Perthes’ disease than the presentation itself. It is well known that preterm children are smaller in size and have a higher incidence of breech presentation. However, we have adjusted for gestational age and birth weight in our analysis, and the risk for Perthes’ disease after birth in breech presentation remains. It could also be hypothesized that breech presentation causes pressure on the hip joint, similar to what is presumed in the case of developmental dysplasia of the hip (DDH) [25]. It could then be plausible for this elevated pressure to alter or damage the circulation of the hip joint. As shown by Kemp, increased intracapsular pressure can cause the changes seen in Perthes’ disease [4]. The question then is, why these changes show much later in life than for example the changes seen in DDH? It could be that there are some immediate changes that we are unable to detect with the methods available today. These changes could then progress as the child grows, remaining undetected until they start giving symptoms.

This study was performed using national registers. There is always a risk that not everything has been correctly registered. Such errors should in this case be random and not systematic. It is possible that not all children with Perthes’ disease are correctly diagnosed, and therefore we could be missing subjects. This number should be very low because of the extensive child health care system in Sweden. We believe that this study is the biggest to date on the effect of pregnancy- and delivery-related factors on the development of Perthes’ disease. Other studies have been conducted on the subject, but these studies range in size from 217 [6] to 852 [4] and thus are all significantly smaller than our study population. All of these studies were conducted using healthy controls, and Wynne-Davies and Gormley used in addition parents and siblings as references for height [6]. An interesting point is that in their 2008 study, Bahmanyar did not find any association between breech presentation and Perthes’ disease [6].

## Conclusion

In conclusion, this study shows that there is an association between a breech position and risk for developing Perthes’ disease. Whether this risk is actually caused by the breech position itself or some of the risk factors for breech position cannot be answered based on this study alone. Despite the significant 25% risk elevation, only approximately 1‰ of children born in breech position develop Perthes’ disease. In Sweden, approximately 3–5% of children are born from a breech presentation. Thus, the number of children with an elevated risk for Perthes’ disease is very small. Should there be some additional controls on breech children? The answer is no, firstly because that would result in numerous unnecessary follow-ups. Secondly, we are not aware of any method or intervention that could prevent the development of Perthes’ disease, or that could be used to diagnose it before skeletal changes are present. We believe that Perthes’ disease may be initiated much earlier than we can diagnose with today’s methods. The disease can also cause great suffering in childhood and lead to debilitating results in adult life because of malformation in the joint. Better understanding of the causes and mechanisms behind its development could lead to earlier diagnosis, better treatment and hopefully prevention.

## Data Availability

The Ethical Review Board approval was obtained for public sharing and presentation of data on group level only. This means that the data used in this study can only be used for the approved research and cannot be shared by the authors.

## Abbreviations

CS: Cesarean section
LGA: Large for gestational age
SGA: Small for gestational age
